# Liver-directed AAV gene therapy in mice corrects glycogen storage disease type IX γ2

**DOI:** 10.1126/sciadv.ady6760

**Published:** 2025-11-12

**Authors:** Rebecca A. Gibson, William R. Jeck, Rebecca L. Koch, Neha Jumani, Su Jin Choi, Deeksha Bali, Sarah P. Young, Aravind Asokan, Jeong-A Lim, Priya S. Kishnani

**Affiliations:** ^1^ Department of Molecular Genetics and Microbiology, Duke University Medical Center, Durham, NC 27705, USA.; ^2^ Division of Medical Genetics, Department of Pediatrics, Duke University Medical Center, Durham, NC 27705, USA.; ^3^ Department of Pathology, Duke University Medical Center, Durham, NC 27705, USA.; ^4^ Department of Surgery, Duke University Medical Center, Durham, NC 27705, USA.

## Abstract

Glycogen storage disease (GSD) type IX γ2 is a rare inborn error of metabolism where a defect in glycogenolysis leads to the inability to break down glycogen in the liver. Patients with GSD IX γ2 develop hypoglycemia and advanced liver disease, placing them at risk for liver transplantation. This study evaluates the efficacy of liver-directed AAV gene therapy in a murine model of GSD IX γ2. *Phkg2^−/−^* mice underwent treatment with AAV gene therapy (AAV9-LSP-*mPhkg2*, 5 × 10^12^ vg/kg, intravenous delivery) at ages 3 or 6 months and were treated for either 2 weeks, 3 months, or 12 months. Results demonstrated that AAV gene therapy reduced GSD IX γ2 disease burden across all primary end points. AAV gene therapy also persisted across the mouse lifespan and reduced preexisting liver fibrosis. This work provides preclinical data supporting AAV gene therapy as a definitive treatment for GSD IX γ2.

## Teaser

AAV gene therapy shows preclinical promise as a definitive treatment for patients with GSD IX γ2.

## INTRODUCTION

Glycogen storage disease type IX (GSD IX) is the most common hepatic GSD, accounting for 25% of all GSD cases with an overall estimated prevalence of 1 in 100,000 individuals (*1*, *2*). Hepatic GSD IX is associated with a deficiency of liver phosphorylase kinase (PhK), a key enzyme in the initiation of liver glycogenolysis (*3*). Liver PhK is a heterotetramer composed of four subunits, α2, β, γ2, and δ, where the α2, β, and δ subunits regulate the activity of the catalytic γ2 subunit (*4*, *5*). Pathogenic variants in the genes encoding the α2, β, and γ2 subunits (*PHKA2*, *PHKB*, and *PHKG2*, respectively) cause liver PhK deficiency, resulting in a diagnosis of hepatic GSD IX (*4*, *5*).

Hepatic GSD IX can be divided into three subtypes based on the defective PhK subunit: GSD IX α2 (historically known as IXa), GSD IX β (IXb), and GSD IX γ2 (IXc) (*1*, *6*). In line with the role of the γ2 subunit as the catalytic domain, GSD IX γ2 is the most severe subtype (*7*, *8*). While all patients with hepatic GSD IX present with similar symptoms, including ketotic hypoglycemia, hepatomegaly, and elevated blood aminotransferase levels, over 95% of patients with GSD IX γ2 develop severe liver disease, including liver fibrosis and cirrhosis, with an increased risk for liver failure (*7*). Despite the progressive liver disease associated with GSD IX γ2, treatment options are limited (*1*, *6*). The current standard of care is dietary management with a high-protein diet and frequent supplementation of uncooked cornstarch to maintain euglycemia (*1*). However, dietary management does not prevent the continued accumulation of glycogen in the liver, which is the underlying pathophysiology of the disease. Therefore, there is a need for a long-term treatment for GSD IX γ2 (*1*, *6*, *9*).

Adeno-associated virus (AAV) vector–based gene therapy is a promising tool for the definitive treatment of GSD IX γ2 (*10*–*18*). There is a well-established clinical precedent for the development of AAV gene therapy for liver-specific monogenic diseases (*19*–*24*), with robust literature demonstrating the preclinical efficacy of AAV gene therapy for GSDs (*25*–*33*). Here, we present evidence for an AAV gene replacement therapy for GSD IX γ2. Our laboratory has recently characterized a previously unknown GSD IX γ2 mouse model (*Phkg2^−/−^*) (*34*, *35*). *Phkg2*^−/−^ mice were injected intravenously via the tail vein with an AAV gene therapy delivering the murine *Phkg2* gene in an AAV9 capsid under the control of a liver-specific promoter (LSP), herein referred to as AAV9-LSP-*mPhkg2*. This work provides preliminary evidence for the efficacy and longevity of AAV gene therapy for GSD IX γ2 and lays the foundation for human clinical trials.

## RESULTS

### *mPhkg2* delivery and expression via AAV gene therapy in GSD IX γ2 mice

**Fig. 1.**
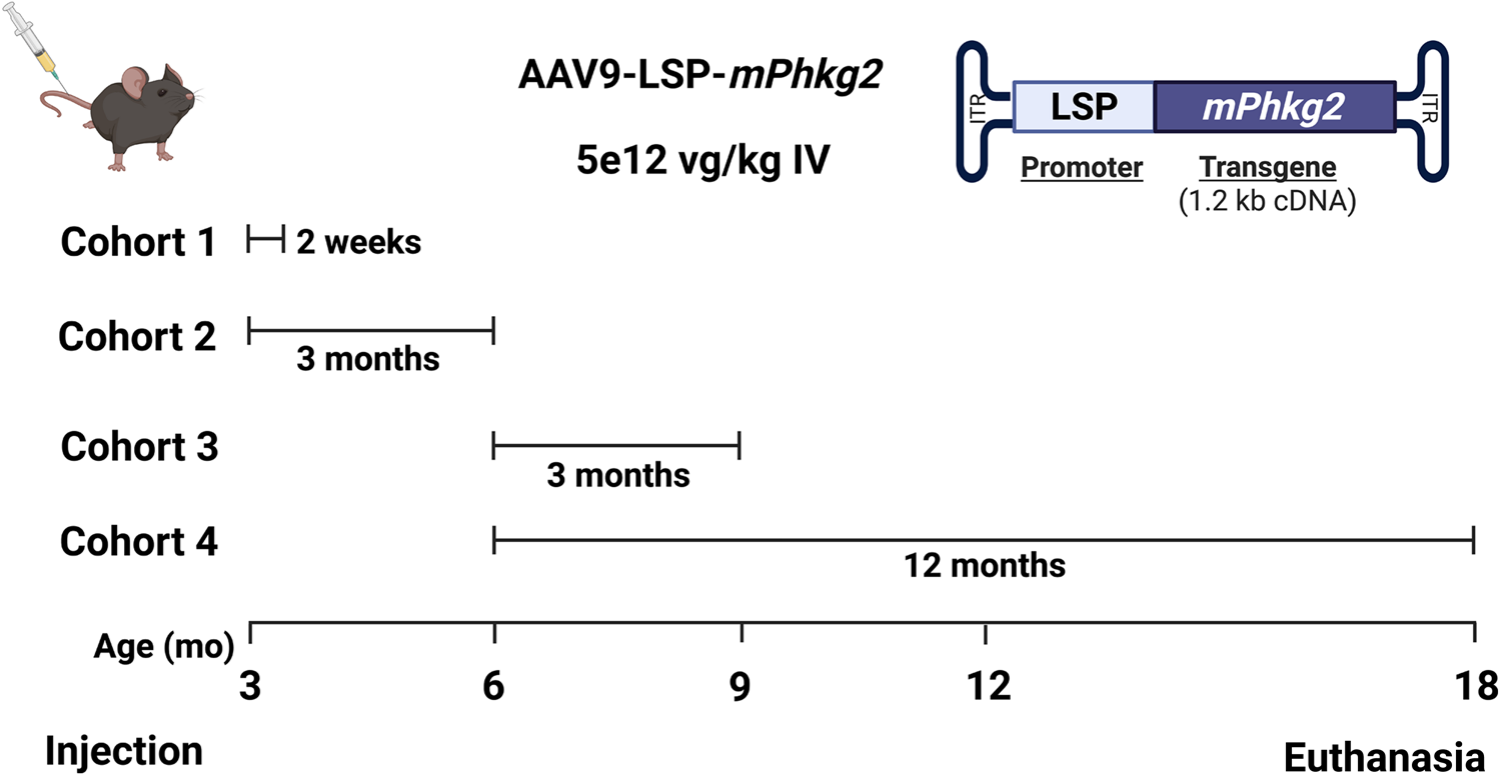
AAV-mediated gene delivery of *Phkg2* to GSD IX γ2 mice. (**A**) Three-month-old (cohorts 1 and 2) and 6-month-old (cohorts 3 and 4) *Phkg2* knockout (*Phkg2^−/−^*, KO) mice were treated with AAV9-LSP-*mPhkg2* at a dose of 5 × 10^12^ vg/kg via tail vein injection. Mice were euthanized at various time points post-treatment: 2 weeks (cohort 1), 3 months (cohorts 2 and 3), and 12 months (cohort 4).

**Fig. 2.**
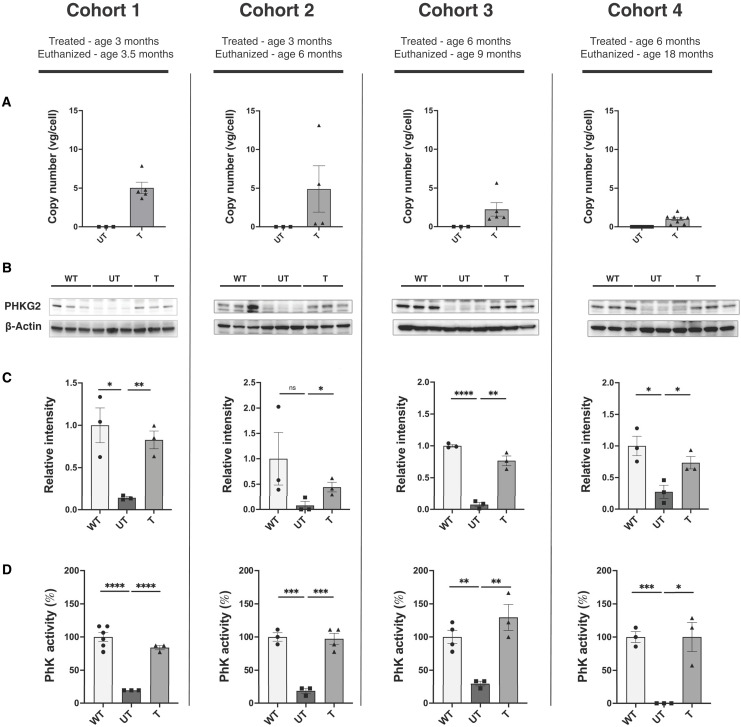
AAV-mediated gene delivery of *Phkg2* to GSD IX γ2 mice. (**A**) AAV genome copy numbers in the liver were quantified by qPCR using *mPhkg2*-specific primers in untreated (UT) and treated (T) mice (*n* = 4 to 8 per group). (**B**) PHKG2 protein levels in the liver were assessed by Western blotting, and β-actin was used as the loading control. (**C**) The relative band intensity of PHKG2 compared to wild-type (WT) controls was quantified and represented in the graphs (*n* = 3 per group). (**D**) Phosphorylase kinase (PhK) enzyme activity was measured in liver lysates (*n* = 3 to 6 per group). Data are presented as mean ± SEM. Statistical significance was determined using an unpaired parametric *t* test, *P < 0.05, **P < 0.01, ***P < 0.001, and ****P < 0.0001.

The murine *Phkg2* cDNA, under the control of an LSP, was packaged into an AAV9 capsid (AAV9-LSP-*mPhkg2*). *Phkg2^−/−^* mice were treated at ages 3 (cohorts 1 and 2) or 6 (cohorts 3 and 4) months at the dose of 5 × 10^12^ vg/kg via tail vein injection for either 2 weeks, 3 months, or 12 months (Fig. 1). Delivery and expression of the PhK γ2 subunit (PHKG2) were confirmed via vector genome quantification, Western blot, and PhK enzyme activity assay. Vector genome quantification indicated successful liver transduction in all cohorts, with the highest quantity in cohort 1 (5.03 ± 1.63 vg/cell) followed by cohort 2 (4.89 ± 5.99 vg/cell), cohort 3 (2.23 ± 1.95 vg/cell), and cohort 4 (1.02 ± 0.59 vg/cell) (Fig. 2A). There was a decline in copy number with treatment age and duration. Western blots confirmed the presence of PHKG2 in all cohorts. PHKG2 level was significantly elevated in treated groups (T) compared to untreated (UT) controls across cohorts (cohort 1, *P* < 0.01; cohort 2, *P* < 0.05; cohort 3, *P* < 0.01; and cohort 4, *P* < 0.05) (Fig. 2, B and C). As PhK is a heterotetrameric enzyme, it was important to ensure that PHKG2 expression restored the functional activity of the PhK complex. PhK enzyme activity was significantly higher in AAV-treated mice compared to UT controls in all four cohorts (cohort 1, *P* < 0.0001; cohort 2, *P* < 0.001; cohort 3, *P* < 0.01; and cohort 4, *P* < 0.05), effectively restoring activity to the wild type (WT) level (Fig. 2D).

### Correction of hepatomegaly and normalization of GSD biomarkers following AAV gene therapy

**Fig. 3.**
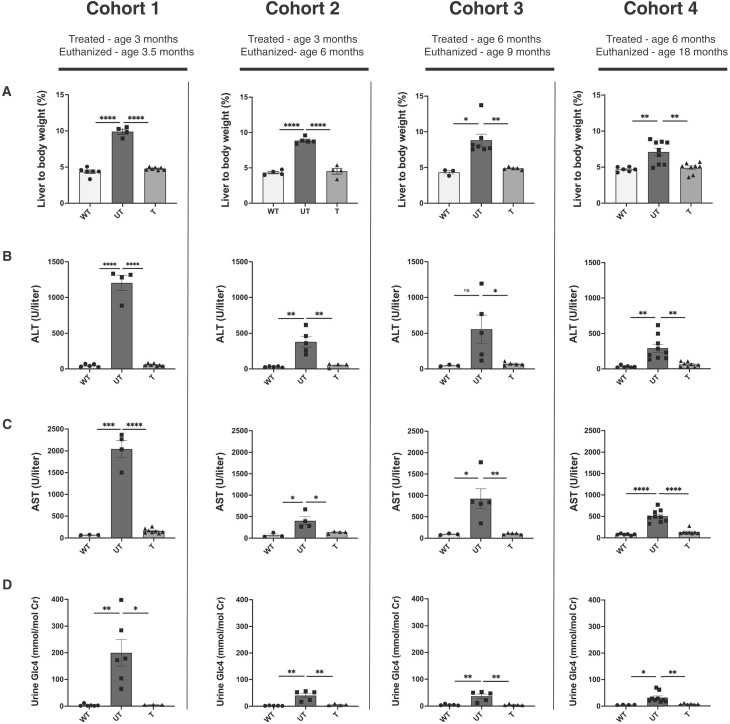
AAV gene therapy alleviates liver abnormalities of GSD IX γ2 in all treated cohorts. (**A**) Liver size expressed as ratio of liver weight to body weight, (**B**) serum alanine aminotransferase (ALT) and (**C**) serum aspartate aminotransferase (AST), and **(D**) urine Glc_4_ levels were significantly decreased in all treated (T) cohorts compared to untreated (UT) mice and were comparable to wild-type (WT) levels. Data are shown as mean ± SEM with *n* = 4 to 9 per group. Statistical significance was determined using unpaired parametric *t* test, *P < 0.05 and **P < 0.01.

**Fig. 4.**
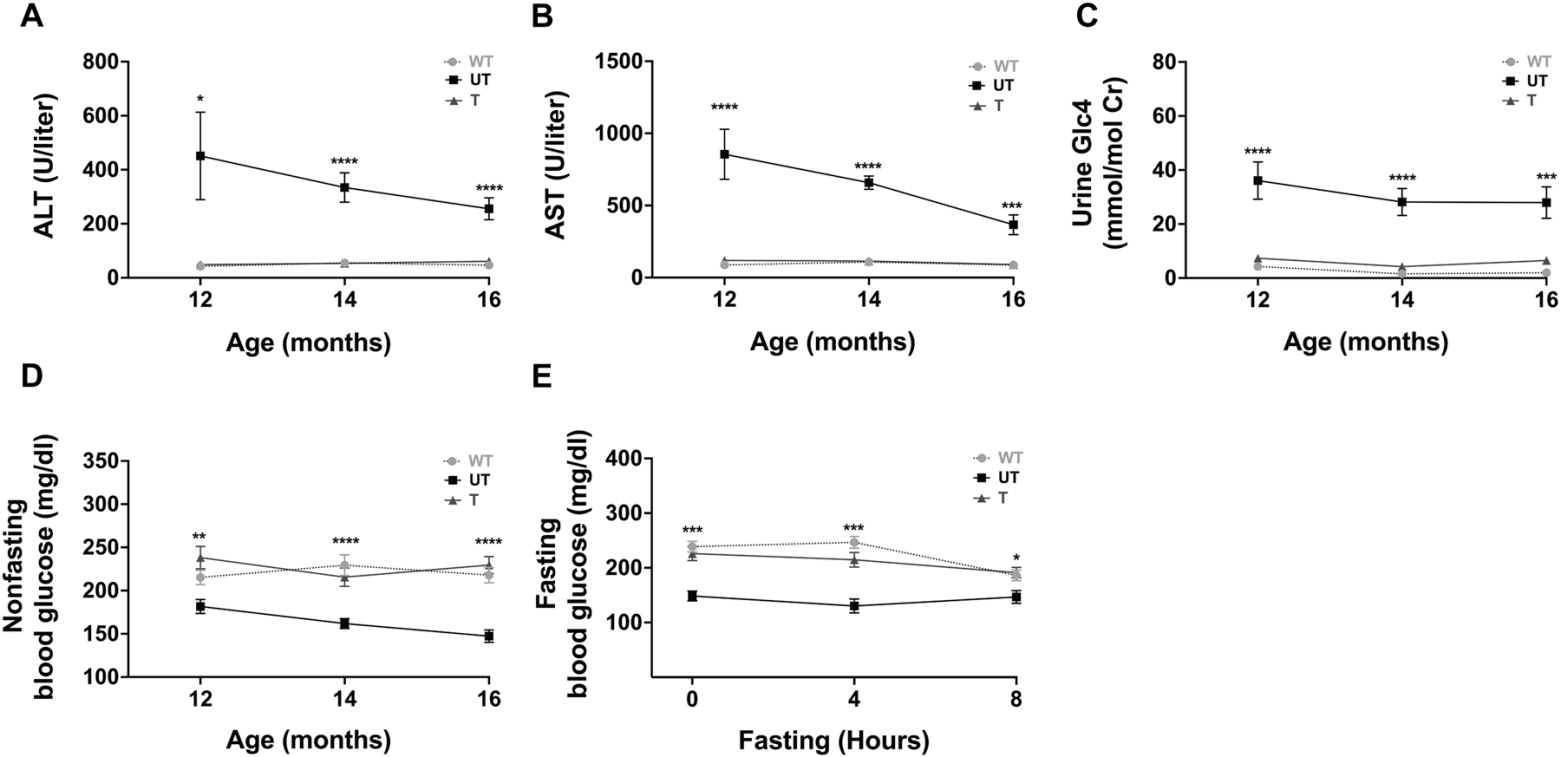
Reduction of liver disease biomarkers and maintenance of euglycemia with long-term AAV treatment. Blood and urine samples were collected from mice in cohort 4 (treated at 6 months of age) at 12, 14, and 16 months of age. (**A**) Serum alanine aminotransferase (ALT), (**B**) serum aspartate aminotransferase (AST), and (**C**) urine glucose tetrasaccharide (Glc_4_) levels were significantly reduced in treated mice compared with untreated (UT) mice and were in the range observed in wild-type (WT) mice. In addition, (**D**) nonfasting blood glucose levels were significantly higher in treated mice over time compared with UT controls and were comparable to WT levels. (**E**) To assess fasting tolerance, mice from cohort 4 at age 18 months were fasted for 8 hours and blood glucose was evaluated at 0, 4, and 8 hours of fasting. Treated mice exhibited significantly increased blood glucose at all time points compared with UT controls, comparable to WT levels. Data shown as mean ± SEM, with *n* = 8 to 9 per group [(A) to (D)] or *n* = 4 to 9 per group (E). Statistical significance was determined using unpaired parametric *t* tests, *P < 0.05, ***P < 0.001, and ****P < 0.0001.

Next, we evaluated whether AAV treatment improved the liver disease phenotype. Liver-to-body weight was significantly reduced in all treatment groups compared to UT controls, and comparable to WT levels (Fig. 3A). Serum alanine aminotransferase (ALT), serum aspartate aminotransferase (AST), and urine glucose tetrasaccharide (Glc_4_) levels were significantly decreased in treated mice in all cohorts compared to UT mice, approaching WT mice levels (Fig. 3, B to D). To assess long-term treatment efficacy of AAV gene therapy for GSD IX γ2, we also evaluated the serum and urine biomarkers in cohort 4 (treated at 6 months of age) at additional time points and showed sustained improvements at 12, 14, and 16 months of age (Fig. 4, A to C). Treatment promoted long-term euglycemia, with normalized nonfasting blood glucose levels in treated mice at all time points (Fig. 4D). In addition, a blood glucose curve from cohort 4 mice at 0, 4, and 8 hours of fasting at 18 months of age showed that treated mice were able to maintain blood glucose levels similar to WT levels and significantly higher than UT levels at all time points (Fig. 4E).

### Reduction of liver glycogen content following AAV gene therapy

**Fig. 5.**
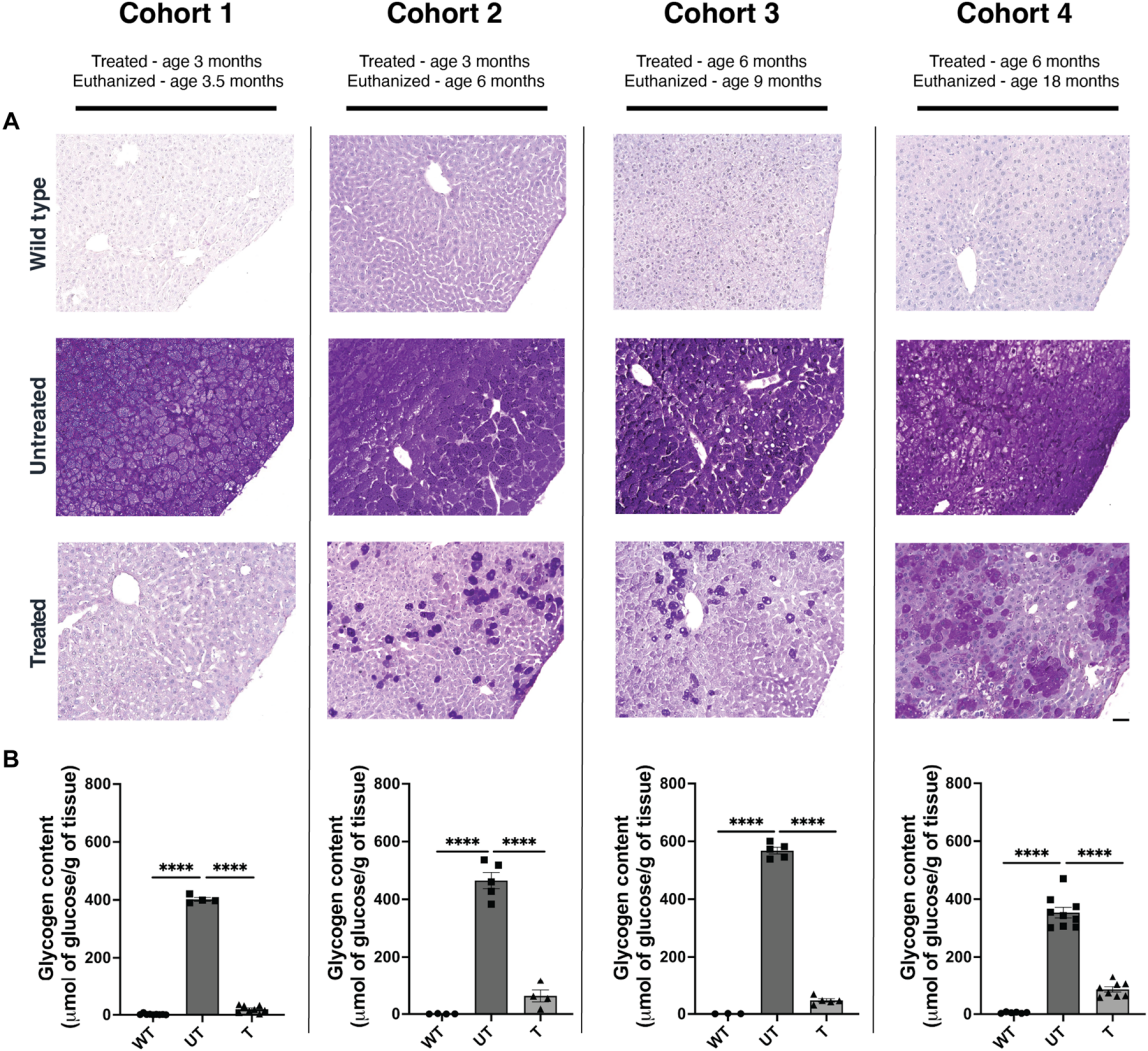
AAV treatment results in reduction of liver glycogen in GSD IX γ2 mice. (**A**) Liver glycogen accumulation was visually evaluated using periodic acid–Schiff (PAS) staining in wild-type (WT), untreated (UT), and treated (T) mice from cohorts 1 to 4. The images presented are representative of *n* = 3 different mice per group. The scale bar represents 50 μm. In addition, (**B**) liver glycogen content was quantitatively measured using a biochemical assay in WT, UT, and T mice from cohorts 1 to 4. Data are presented as mean ± SEM, with *n* = 4 to 9 per group. Statistical significance was determined using unpaired parametric *t* tests, **** *P* < 0.0001.

We assessed the amount of glycogen in the livers of treated, UT, and WT mice using periodic acid–Schiff (PAS) staining and biochemical assay. PAS stain showed a remarkable reduction of liver glycogen in treated mice across all cohorts; however, a slight increase in glycogen content was observed in treated mice from cohort 1 to cohort 4, with increased positive staining relative to WT controls (Fig. 5A). A blinded hepatopathologist confirmed a substantial reduction in glycogen content with treatment (table S3). Biochemical evaluation of liver glycogen further demonstrated significantly reduced liver glycogen content in treated mice in all cohorts (cohorts 1 to 4, *P* < 0.0001), with a slight increase above WT as treatment duration increased (Fig. 5B).

### Restoration of hepatocyte architecture with reversal of fibrosis following AAV gene therapy

**Fig. 6.**
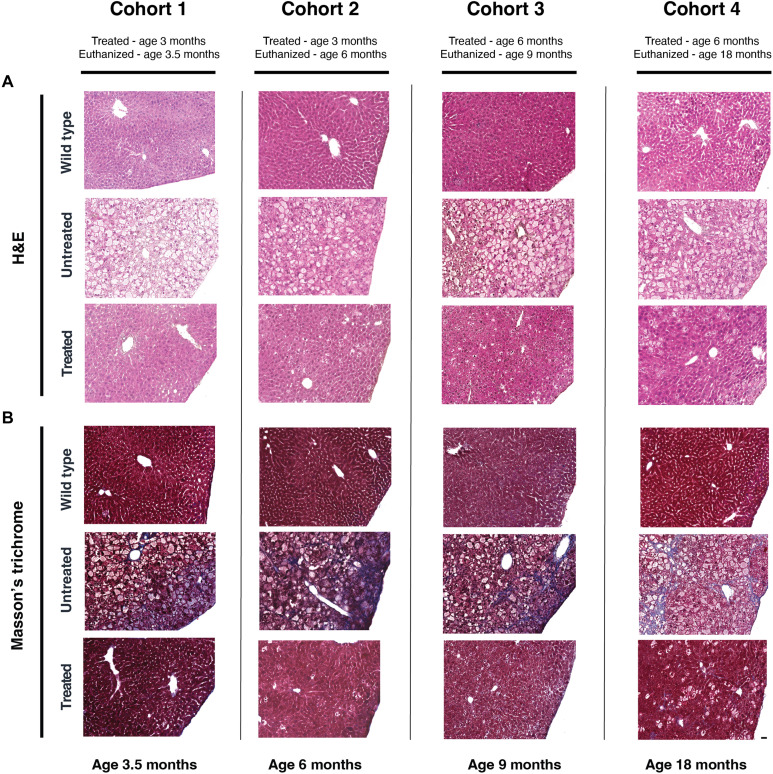
AAV gene therapy corrects hepatocyte architecture and reduces fibrosis in GSD IX γ2 mice. Paraffin-embedded liver sections were stained with (**A**) hematoxylin and eosin (H&E) to evaluate hepatocyte architecture, as well as (**B**) Masson’s trichrome to evaluate liver fibrosis, with blue staining indicating fibrosis, in wild-type (WT), untreated (UT), and treated (T) mice from cohorts 1 to 4. Representative images from three to five mice per group are shown. The scale bar represents 50 μm.

Hematoxylin and eosin (H&E) and Masson’s trichrome staining of the liver demonstrated restoration of hepatocyte architecture and reversal of liver fibrosis in all treated cohorts (Fig. 6 and tables S4 to S6). H&E staining also confirmed there was no inflammation associated with treatment (table S5) and demonstrated a reduction in hepatocyte size and restoration of nuclear morphology in treated mice (table S6). Hepatocellular carcinoma was not observed in any study animal. Moreover, the scoring of trichrome-stained slides confirmed a significant reduction in liver fibrosis in all treated mice, when compared with UT and WT controls (Table 1). In cohort 1, treated mice had an average fibrosis score approximately 3× lower than UT controls (treated 1.0 ± 1.0 and UT 2.7 ± 0.6). In cohorts 2 and 3, treated mice had an average fibrosis score approximately 1.5× lower than UT controls (cohort 2: treated 1.5 ± 1.0 and UT 3.0 ± 0.0; cohort 3: treated 1.8 ± 0.4 and UT 2.6 ± 0.5). Last, treated mice from cohort 4 had an average fibrosis score approximately 2.5× lower than UT controls (treated 2.3 ± 0.9 and UT 4.9 ± 1.3) (Table 1).

**Table 1.** AAV gene therapy reduces liver fibrosis in all cohorts. NA, not available.

		Fibrosis score (0–6)		
		Average	Range	*P* value
**Cohort 1:** Treated, age 3 months; euthanized, age 3.5 months	**WT**	0	NA	0.0286
	**Untreated**	3	2–3	
	**Treated**	1	0–2	
**Cohort 2:** Treated, age 3 months; euthanized, age 6 months	**WT**	0	NA	0.0016
	**Untreated**	3	NA	
	**Treated**	2	0–2	
**Cohort 3:** Treated, age 6 months; euthanized, age 9 months	**WT**	1	0–1	0.0014
	**Untreated**	3	3–6	
	**Treated**	2	1–4	
**Cohort 4:** Treated, age 6 months; euthanized, age 18 months	**WT**	0	0–1	<0.0001
	**Untreated**	5	3–6	
	**Treated**	2	1–4	

### Restoration of glycogen metabolism following AAV gene therapy

**Fig. 7.**
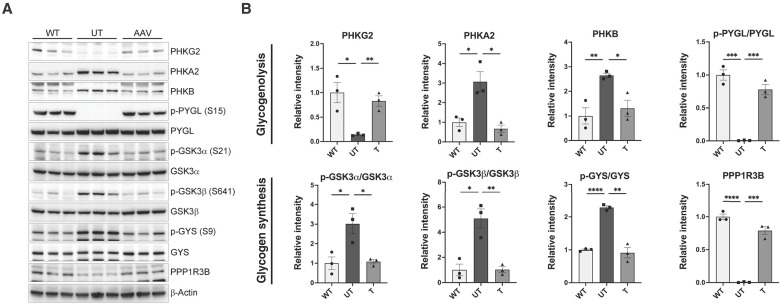
AAV gene therapy restores glycogen metabolism enzyme expression in cohort 1 treated mice. (**A**) The expression and phosphorylation of key glycogen metabolism proteins in liver samples from treated (T) mice in cohort 1 were evaluated by Western blot using specific antibodies. Age-matched wild-type (WT) and untreated (UT) mice served as controls. β-Actin was used as the loading control. (**B**) Western blot band intensities were quantified, normalized to β-actin, and expressed as a relative intensity compared to WT levels. Data are shown as mean ± SEM, with *n* = 3 per group. Statistical significance was determined using unpaired parametric *t* tests, *P < 0.05, **P < 0.01, and ***P < 0.001.

Last, given the known glycogen metabolism perturbations in the *Phkg2^−/−^* model (*35*), we investigated key proteins involved in glycogen metabolism in mice from cohort 1 (treated at 3 months of age, evaluated 2 weeks later) to assess whether the abnormalities in glycogen metabolism were mitigated by AAV treatment. We measured levels of PHKG2, PHKA2, PHKB, phosphorylated and total PYGL, phosphorylated and total GSK3α, phosphorylated and total GSK3β, phosphorylated and total glycogen synthase, and PPP1R3B. AAV gene therapy treatment significantly restored all protein expression levels in treated mice compared with UT controls, comparable to WT levels (Fig. 7).

## DISCUSSION

GSD IX γ2 is a severe liver disease caused by PhK deficiency, resulting in disruptions to glycogenolysis, hypoglycemia, accumulation of liver glycogen, and progressive liver fibrosis or cirrhosis (*7*, *8*, *36*–*38*). Despite the life-threatening severity of advanced liver disease in GSD IX γ2, there are currently no definitive treatments available for patients.

Our laboratory previously characterized a previously unknown GSD IX γ2 mouse model (*Phkg2^−/−^*) (*34*, *35*). We demonstrated an early liver disease phenotype in GSD IX γ2 mice, with substantial disease progression. GSD IX γ2 mice up to 24 months of age demonstrated hepatomegaly, hypoglycemia, and elevated liver glycogen content, which progressed to severe liver fibrosis with cirrhotic nodule formation, and a concomitant increase and then decrease in serum and urine GSD biomarkers (*34*, *35*). We see a similar pattern in our patients with GSD IX γ2 (*1*, *7*). The in-depth characterization of the GSD IX γ2 mouse model provided a platform for the evaluation of potential GSD IX γ2 therapeutics.

Our study provides compelling evidence demonstrating the efficacy of AAV gene therapy as a long-term, noninvasive, and definitive treatment for GSD IX γ2. As a monogenic disease with a liver-specific phenotype, GSD IX γ2 was an ideal candidate for treatment with AAV gene therapy. With the liver being the primary organ affected in GSD IX γ2, we used a LSP that not only targets the liver but also has a known effect of inducing immune tolerance to the transgene, allowing for efficacy at lower dosing (*39*, *40*). Treatment with our AAV gene therapy construct proved effective across the mouse lifespan (up to 18 months of age), an important finding as it is well established that liver-directed AAV gene therapy can have a dilutional effect in the liver (*41*–*43*). In comparison to viral vectors that integrate into the host genome, AAV genomes exist as episomes and are prone to dilution with natural hepatocyte regeneration (*44*). There was an expected gradual reduction in vector genome copy number between cohorts 1 and 2 (treated at 3 months of age) and cohorts 3 and 4 (treated at 6 months of age)**,** potentially due to the higher burden of liver fibrosis at injection at older ages. Despite the differences in vector genome copies, our AAV gene therapy construct had proven efficacy across all cohorts, with protein expression and PhK enzyme activity restored in all cohorts (Fig. 2). The liver size, serum and urine biomarkers, and liver glycogen content were all significantly reduced across cohorts (Fig. 3 to 5). Our data show that our construct can demonstrate therapeutic efficacy for GSD IX γ2 mice at a 5 × 10^12^ vg/kg dose, and that efficacy is demonstrated over time. Previous work with AAV gene therapy in other GSD murine models has demonstrated that small percentages of restored enzyme activity are needed to correct GSD phenotypes. In previous studies with GSD Ia mice, restoring 0.5 to 3% of WT enzyme activity across 70 to 90 weeks corrected blood biomarkers (*45*, *46*). In our study, AAV gene therapy across 48 weeks provided an average of 99% WT enzyme activity levels (Fig. 2D) with normalization of blood and urine biomarkers as well as reduced hepatic glycogen levels, providing encouraging evidence that despite the dilutional effect of hepatocyte turnover, AAV gene therapy can have marked efficacy over time.

With increased risk for hypoglycemia and other metabolic abnormalities, patients with GSD IX γ2 are recommended to follow a high-protein diet with frequent supplementation of uncooked cornstarch. The risk of hypoglycemia in patients with GSD IX γ2 often results in the parents and/or patient themselves having to wake up overnight to get a bolus dose of cornstarch (*1*). Our previous work found that *Phkg2^−/−^* mice are hypoglycemic in the early stages of fasting compared to WT controls (*34*, *35*). In this study, blood glucose levels in mice treated with AAV gene therapy were higher than UT mice and comparable to WT controls, suggesting that treatment allows GSD IX γ2 mice to maintain normoglycemia. Moreover, fasting blood glucose curves indicated that AAV-treated mice maintained normoglycemia during prolonged fasting without overcorrecting to hyperglycemia (Fig. 4). Studies will be needed to assess if these findings result in a discontinuation or reduction in patient consumption of cornstarch supplementation (*47*).

Previous preclinical studies have established the efficacy of AAV gene therapy in expressing transgene in animal models of inborn errors of metabolism that develop liver fibrosis, including models of other GSDs (*48*, *49*). For instance, in a mouse model of GSD IIIa, treatment with AAV gene therapy reversed and prevented early-stage liver fibrosis in 5-month-old mice (*28*, *30*). Similarly, our study demonstrated that AAV gene therapy can reverse and/or prevent fibrosis in GSD IX γ2 mice. In Gibson *et al.* (*35*), we introduced a novel scoring system created to evaluate liver fibrosis severity specific to the *Phkg2^−/−^* mouse model. From this scoring system, it is known that GSD IX γ2 mice at 3 months of age display minimal subcapsular pericellular fibrosis and at 6 months of age display subcapsular pericellular fibrosis (*34*, *35*). When GSD IX γ2 mice were treated at 3 months of age and euthanized after 2 weeks and 3 months (cohorts 1 and 2, respectively), there was a marked prevention in the progression of liver fibrosis. Even when GSD IX γ2 mice were treated at 6 months and euthanized after 12 months (cohort 4), the fibrosis score for treated mice at age 18 months was lower than the fibrosis score of mice before initial treatment (cohort 1), demonstrating that AAV gene therapy in GSD IX γ2 mice can markedly reverse liver fibrotic burden. Previous studies have confirmed that with the removal of the source of chronic liver injury, in this case, chronic glycogen buildup, liver fibrosis can regress in animal models and patients (*48*, *50*). In our study, a significant reduction of glycogen accumulation was observed across all treated cohorts, and this reduction correlated with the prevention and reversal of the progression of liver fibrosis following AAV gene therapy, without increased hepatocyte inflammation (Figs. 5 and 6 and table S5). Our work demonstrates a reversal of fibrosis despite different stages of preexisting liver disease, a therapeutic effect needed for the transition toward clinical trial. Future work demonstrating therapeutic efficacy at later stages of disease (greater than age 6 months) could provide additional support for clinical trial participants at different stages of disease progression.

Last, our study demonstrates that GSD IX γ2 AAV gene therapy corrects the PhK enzyme complex, providing restoration of glycogen metabolism pathways. As previously described, GSD IX γ2 mice demonstrate a general up-regulation of glycogenolysis and down-regulation of glycogenesis enzymes (*35*). AAV gene therapy restored glycogenolysis and glycogenesis enzyme expression in treated mice to WT levels (Fig. 7). As PhK is a heterotetramer, with γ2 as the enzymatic catalyst, restoration of glycogen metabolism supports correction of the PhK complex, allowing the PhK heterotetramer to initiate the subsequent enzymatic steps of glycogenolysis. Our data also provide preclinical evidence toward glycogen metabolism as a primary disease end point in future GSD gene therapy clinical trials.

In conclusion, our study provided evidence of the efficacy of AAV gene therapy for the treatment of GSD IX γ2. This therapy effectively increased PhK enzyme activity, reduced liver glycogen, decreased serum and urine biomarkers, and promoted euglycemia. AAV gene therapy demonstrated prolonged benefits across the mouse lifespan without tumor formation or increased hepatocyte inflammation. In addition, AAV gene therapy effectively prevented and reversed liver fibrosis progression. Restoration of the glycogen metabolism pathways supported correction of the PhK enzyme’s function in glycogenolysis and serves as an additional end point for the evaluation of AAV therapeutic efficacy in patients. Our study provides ample evidence to translate our therapeutic platform to other GSD IX subtypes and progress our AAV construct for GSD IX γ2 toward clinical trial. Future work evaluating dose optimization and immune response in larger animal models will be needed to better understand dose efficacy over time.

## MATERIALS AND METHODS

### AAV vector constructs and packaging

To generate the pAAV-CB-*mPhkg2* plasmid, we cloned the 1.2-kb murine *Phkg2* cDNA (NM_026888.3) from the KpnI-EcoRI fragment from the pcDNA3.1mPhkg2 plasmid (GenScript, Piscataway, NJ) into the pAAV-CB-hGAA vector containing the CMV enhanced chicken β-actin hybrid (CB) promoter to replace the human *GAA* cDNA (*51*). To generate the pAAV-LSP-*mPhkg2* plasmid, we cloned the ScaI-KpnI fragment containing the LSP from the pAAV-LSP-hGAA vector into the pAAV-CB- *mPhkg2* vector to replace the CB promoter (*39*). This plasmid was packaged into AAV9 (AAV9-LSP-*mPhkg2*) in human embryonic kidney 293T cells using the calcium phosphate transfection method and purified using the iodixanol gradient ultracentrifugation method as previously described (*30*, *52*). The titer of the viral stock was determined using quantitative polymerase chain reaction (qPCR) with primers against the *mPhkg2* transgene (table S1). The viral vector stock was handled according to biohazard safety level 2 guidelines published by the National Institutes of Health (*39**,* *51**,* *52*).

### Study design

GSD IX γ2 mice (*Phkg2^−/−^*) were bred and genotyped as described (*34*). Four cohorts of GSD IX γ2 mice were injected with AAV9-LSP-*mPhkg2* at a dose of 5 × 10^12^ vg/kg via the tail vein. The first two cohorts (cohorts 1 and 2) were injected at 3 months of age, and the other two cohorts (cohorts 3 and 4) were injected at 6 months of age, evaluating treatment at different stages of disease progression. Age-matched UT *Phkg2^−/−^* mice and WT *Phkg2^+/+^* mice were used in all cohorts as controls. All mice were fasted for 24 hours before euthanasia. Mice were euthanized at 2 weeks, 3 months, or 12 months postinjection to evaluate treatment duration (Fig. 1). Body weight was measured, and blood and urine samples were collected. The whole liver was collected and weighed to evaluate the ratio of liver weight to body weight. Samples of liver tissue were then frozen on dry ice and stored at −80°C for biochemical assays or fixed in 10% neutral-buffered formalin (NBF) for 48 hours for histological analysis. The primary end points for the experiment were vector genome quantification, PHKG2 expression, PhK enzyme activity, liver–to–body weight ratio, serum aminotransferase (ALT and AST) levels, urine Glc_4_ levels, glycogen content, hepatocyte architecture, presence of liver fibrosis, and expression of proteins involved in glycogen metabolism. All animal experiments were performed under the approval of the Duke University Institutional Animal Care and Use Committee (IACUC no. A170-21-08) and the guidelines from the National Institute of Health guide for the care and use of laboratory animals (*53*).

### AAV vector genome quantification

AAV vector genomes were quantified by qPCR as previously described (*28*). In brief, the linearized pAAV-LSP-*mPhkg2* plasmid DNA was used to generate a standard curve for calculating viral vector copy numbers. Genomic DNA was extracted from frozen tissues using the Wizard Genomic DNA Purification kit (Promega, Madison, WI). PCR was performed using SYBR Green (Bio-rad, Hercules, CA) and the gene-specific primer pairs for *mPhkg2* and mouse β-actin (table S1) (*28*).

### Western blot

Western blots were performed as previously described (*34*). Tissues were homogenized in cold RIPA buffer containing a protease/phosphatase inhibitor (Cell Signaling Technology, Danvers, MA). Homogenization was performed using an electric homogenizer. Centrifugation was performed at 18,000*g* at 4°C for 15 min to obtain clear tissue lysates. BCA assay was used to measure the protein concentrations. SDS–polyacrylamide gel electrophoresis gels were used to separate out equal amounts of protein, which were then transferred to nitrocellulose membranes. The membranes were blocked in 3% bovine serum albumin/phosphate-buffered saline with Tween 20 (PBST). Membranes were incubated with primary antibodies at 4°C overnight. Then, the membranes were washed, incubated with secondary antibodies (table S2), and washed again. Enhanced chemiluminescence (ECL) kit (Bio-rad, Hercules, CA) was then used to develop the membranes. Images were obtained using an image analyzer (Bio-rad, Hercules, CA). Images were later quantified using ImageJ (National Institutes of Health, Bethesda, MD), normalized to β-actin, and adjusted for nonspecific background banding.

### PhK enzyme activity assay

PhK enzyme activity assays were performed at the Duke University Health System Biochemical Genetics Laboratory. Assays were performed based on the laboratory’s previously described spectrophotometric methods (*34*). In brief, liver tissue was shipped on dry ice to the biochemical genetics laboratory. Upon arrival, the liver tissues were disrupted, and then inactive phosphorylase b in the liver lysate was converted to active phosphorylase a. Next, glycogen was broken down by the active phosphorylase to release the free glucose molecule in the form of glucose-1-phosphate (G1P). Subsequently, G1P was converted to glucose-6-phosphate (G6P) by phosphoglucomutase (Sigma-Aldrich Co., St. Louis, MO). G6P was oxidized, and nicotinamide adenine dinucleotide (oxidized form) (NAD^+^) was converted to NADH (reduced form of NAD^+^) by G6P dehydrogenase in the Glucose (Hexokinase) Liquid Reagents (Pointe Scientific, Fisher, Hampton, NH). The amount of NADH formed, which is proportional to the concentration of glucose in the sample, was measured by absorbance at 340 nm using a UV-VIS spectrophotometer (Shimadzu UV-1700 PharmaSpec). Enzyme activity was expressed as μmol/min/mg of liver tissue (*36**,* *54*).

### Glycogen content assay

Glycogen content assay was performed on the basis of the previously described methodology (*29*, *34*). Frozen liver tissues were homogenized in distilled water (1 mg of tissue per 20 μl of distilled water). Homogenization was performed using an electric homogenizer. Sonication was performed for 15 s, followed by centrifugation for 15 min at 18,000*g* at 4°C. To inactivate endogenous enzymes, lysates were then boiled for 3 min. Samples were incubated with amyloglucosidase at 0.175 U/ml (Sigma-Aldrich Co., St. Louis, MO) at 37°C for 1.5 hours to release free glucose. To stop the reaction with amyloglucosidase, the mixtures were then boiled for 3 min. Thirty microliters of the mixtures were incubated with 1 ml of Pointe Scientific Glucose Hexokinase Liquid Reagent (Fisher, Hampton, NH) to convert glucose to G6P. Then, the amount of NADH was measured as described previously.

### Histology

Histology was performed with PAS staining to evaluate glycogen content, H&E to evaluate hepatocyte architecture, and Masson’s trichrome to evaluate liver fibrosis based on previously described methodology (*34*, *35*). First, fresh tissue samples were fixed in 10% NBF for 48 hours. Samples were then postfixed with 1% periodic acid (PA) in 10% NBF for 48 hours at 4°C. Samples were then washed with PBS. Samples were dehydrated with ascending grades of alcohol. Samples were then cleared using xylene. Samples were then embedded with paraffin. Sections were cut with a microtome and then mounted in preparation for staining. Staining methodology has been previously described (*34*, *35*). All images were taken using a BZ-X710 microscope (Keyence America, Itasca, IL). The slides were reviewed by a board-certified hepatopathologist with expertise in glycogen storage diseases. The reviewer was blinded to genotype, treatment group, and age. The slides were reviewed and scored on the basis of a previously established scoring system created to evaluate liver fibrosis severity specific to the GSD IX γ2 mouse model (*35*). In addition, the slides were also reviewed for glycogen content, inflammation, nuclear morphology, hepatocyte morphology, and the presence of steatosis.

### Serum and urine biomarkers

Blood was collected in a red top collection tube (BD Biosciences, Franklin Lakes, NJ), incubated for at least 30 min at room temperature and then centrifuged at 2000*g* at 4°C for 10 min to isolate serum. Serum ALT and AST levels were measured using the Pointe Scientific, Liquid ALT Reagent Set A7526 as previously described (*55*). To evaluate nonfasted blood glucose, whole blood was evaluated using the AlphaTRAK2 whole blood glucometer (Zoetis, Durham, NC). To create a blood fasting blood glucose curve, whole blood was sampled at 0, 4, and 8 hours of fasting and measured for blood glucose using the AlphaTRAK2 glucometer (Zoetis, Durham, NC) (*56*). For urine collection, urine was sampled after 24 hours of fasting. Mice were restrained and placed over a collection device. To induce urination, gentle pressure was placed on the belly. Approximately 50 μl of urine was collected from each mouse in individual 1.7-ml Eppendorf tubes. Urine samples were shipped on dry ice to the Duke University Health System Biochemical Genetics Laboratory for urine glucose tetrasaccharide Glcα1-6Glcα1-4Glcα1-4Glc (Glc_4_) analysis. Samples were analyzed based on previously described methods (*57**,* *58*).

### Statistical analysis

Statistical analysis was performed using Prism software version 10 (GraphPad, La Jolla, CA). For quantitative data, statistical analysis was performed using Kruskal Wallis, ordinary one-way analysis of variance (ANOVA), and parametric unpaired *t* tests to determine the differences between wild-type, untreated, and treated groups. *P* value <0.05 was considered statistically significant. P values were characterized using the following system: *P < 0.05, **P < 0.01, ***P < 0.001, and ****P < 0.0001.
