# Supplementary material for: Liver-directed AAV gene therapy in mice corrects glycogen storage disease type IX γ2
Source: Sci Adv. 2025 Nov 12;11(46):eady6760. doi: 10.1126/sciadv.ady6760 (PMC12609164; doi:10.1126/sciadv.ady6760)
Supplement: Supplementary file 1 — Tables S1 to S6 [file sciadv.ady6760_sm.pdf]

Science Advances
AAAS

Supplementary Materials for

Liver-directed AAV gene therapy in mice corrects glycogen storage disease type IX γ2

Rebecca A. Gibson *et al.*

Corresponding author: Jeong-A Lim, jeonga.lim@duke.edu; Priya S. Kishnani, priya.kishnani@duke.edu

Sci. Adv. **11**, eady6760 (2025)
DOI: 10.1126/sciadv.ady6760

This PDF file includes:

Tables S1 to S6

Primers			
**Target**	**Direction**	**Name**	**Sequence**
mPhkg2	Forward	mPhkg2-F	GAGATGCACATTCTTCGCCA
	Reverse	mPhkg2-R	TCCTTTCCGCATCAGGTCAAA
β-Actin	Forward	Actin-F	AGATGTGGATCAGCAAGCAG
	Reverse	Actin-R	GCGCAAGTTAGGTTTTGTCA

Table S1.
PCR primers used for vector genome quantification.

Antibodies				
**Target**	**Size (kDa)**	**Company**	**Catalog #**	**Location**
PHKG2	46	Proteintech	15109-1-AP	Rosemont, Illinois, USA
PHKA2	138	Proteintech	24658-1-AP	Rosemont, Illinois, USA
PHKB	124	Proteintech	13400-1-AP	Rosemont, Illinois, USA
p-PYGL (S15)	97	Abcam	ab227043	Cambridge, UK
PYGL	97	Proteintech	15851-1-AP	Rosemont, Illinois, USA
p-GSK3α (Ser21)	51	Cell-Signaling	9316	Danvers, Massachusetts, USA
GSK3α	51	Cell-Signaling	4337	Danvers, Massachusetts, USA
p-GSK3β (Ser9)	46	Cell-Signaling	9323s	Danvers, Massachusetts, USA
GSK3β	46	Cell-Signaling	9315s	Danvers, Massachusetts, USA
p-GS (Ser641)	85-90	Cell-Signaling	3891s	Danvers, Massachusetts, USA
GS	85-90	Cell-Signaling	3886	Danvers, Massachusetts, USA
PPP1R3B	31	Aviva Systems Bio	ARP61962_P050	San Diego, California, USA
β-Actin-HRP	42	Sigma-Aldrich	A3854	St. Louis, Missouri, USA
anti-Rabbit IgG (whole molecule)-Peroxidase	NA	Sigma-Aldrich	A0545	St. Louis, Missouri, USA

Table S2.
Antibodies used for Western Blot.

		Glycogen Content			
		Approximate Glycogenated Cells (0-100%)	Average	Range	P-value (<0.05)
Cohort 1	Treated	1	1	1	** 0.0036
	Treated	1			
	Treated	1			
	Untreated	100	100	100	
	Untreated	100			
	Untreated	100			
	Wild Type	0	0	0	
	Wild Type	0			
	Wild Type	0			
Cohort 2	Treated	30	28	5-60	*** 0.0003
	Treated	15			
	Treated	60			
	Treated	5			
	Untreated	100	100	100	
	Untreated	100			
	Untreated	100			
	Untreated	100			
	Wild Type	0	0	0	
	Wild Type	0			
	Wild Type	0			
Cohort 3	Treated	10	18	10-25	**** <0.0001
	Treated	20			
	Treated	25			
	Treated	25			
	Treated	10			
	Untreated	100	100	100	
	Untreated	100			
	Untreated	100			
	Untreated	100			
	Untreated	100			
	Wild Type	0	0		
	Wild Type	0			
	Wild Type	0			
Cohort 4	Treated	40	43	25-70	**** <0.0001
	Treated	40			
	Treated	45			
	Treated	30			
	Treated	45			
	Treated	70			
	Treated	50			
	Treated	25			
	Untreated	100	100	100	
	Untreated	100			
	Untreated	100			
	Untreated	100			
	Untreated	100			
	Untreated	100			
	Untreated	100			
	Untreated	100			
	Untreated	100			
	Wild Type	0	0	0	
	Wild Type	0			
	Wild Type	0			
	Wild Type	0			
	Wild Type	0			

Table S3.

AAV gene therapy reduces liver glycogen content in treated mice.

		Fibrosis				
		**Description**	**Score (0-6)**	**Average**	**Range**	**P-value (<0.05)**
**Cohort 1**	Treated	None	0	1	0-2	* 0.0286
	Treated	Focal subcapsular pericellular fibrosis	1			
	Treated	Multifocal subcapsular pericellular fibrosis	2			
	Untreated	Abundant subcapsular pericellular fibrosis	3	3	2-3	
	Untreated	Abundant subcapsular pericellular fibrosis	3			
	Untreated	Multifocal subcapsular pericellular fibrosis	2			
	Wild Type	None	0	0		
	Wild Type	None	0			
	Wild Type	None	0			
**Cohort 2**	Treated	None	0	2	0-2	** 0.0016
	Treated	Multifocal subcapsular pericellular fibrosis	2			
	Treated	Multifocal subcapsular pericellular fibrosis	2			
	Treated	Multifocal subcapsular pericellular fibrosis	2			
	Untreated	Abundant subcapsular with focal bridging	3	3		
	Untreated	Abundant subcapsular with focal bridging	3			
	Untreated	Abundant subcapsular pericellular fibrosis	3			
	Untreated	Abundant subcapsular pericellular fibrosis	3			
	Wild Type	None	0	0		
	Wild Type	None	0			
	Wild Type	None	0			
**Cohort 3**	Treated	Focal subcapsular pericellular fibrosis	1	2	1-2	** 0.0014
	Treated	Multifocal subcapsular pericellular fibrosis	2			
	Treated	Multifocal subcapsular pericellular fibrosis	2			
	Treated	Multifocal subcapsular pericellular fibrosis	2			
	Treated	Multifocal subcapsular pericellular fibrosis	2			
	Untreated	Abundant subcapsular pericellular fibrosis	3	3	2-3	
	Untreated	Abundant subcapsular pericellular fibrosis	3			
	Untreated	Multifocal subcapsular pericellular fibrosis	2			
	Untreated	Abundant subcapsular with focal bridging	3			
	Untreated	Multifocal subcapsular pericellular fibrosis	2			
	Wild Type	Focal subcapsular pericellular fibrosis	1	1	0-1	
	Wild Type	Focal subcapsular pericellular fibrosis	1			
	Wild Type	None	0			
**Cohort 4**	Treated	Focal subcapsular pericellular fibrosis	1	2	1-4	****<0.0001
	Treated	Focal bridging fibrosis	4			
	Treated	Multifocal subcapsular fibrosis	2			
	Treated	Multifocal subcapsular fibrosis	2			
	Treated	Multifocal subcapsular fibrosis	2			
	Treated	Multifocal subcapsular fibrosis	2			
	Treated	Multifocal subcapsular fibrosis, isolated to liver tip	2			
	Treated	Abundant subcapsular pericellular fibrosis	3			
	Untreated	Bridging	5	5	3-6	
	Untreated	Subcapsular fibrosis with multifocal bridging and nodule	6			
	Untreated	Subcapsular fibrosis with multifocal bridging and nodule	6			
	Untreated	Subcapsular fibrosis with multifocal bridging and nodule	6			
	Untreated	Abundant subcapsular pericellular fibrosis	3			
	Untreated	Subcapsular fibrosis with focal bridging	4			
	Untreated	Abundant subcapsular pericellular fibrosis	3			
	Untreated	Subcapsular fibrosis with multifocal bridging and nodule	6			
	Untreated	Subcapsular fibrosis with multifocal bridging	5			
	Wild Type	None	0	0	0-1	
	Wild Type	None	0			
	Wild Type	None	0			
	Wild Type	None	0			
	Wild Type	Focal subcapsular pericellular fibrosis	1			

Table S4.

AAV gene therapy reduces liver fibrosis in treated mice.

		Inflammation				
		Description	Score (0-3)	Average	Range	P-value (<0.05)
Cohort 1	Treated	None	0	0	0	>0.9999
	Treated	None	0			
	Treated	None	0			
	Untreated	None	0	0		
	Untreated	None	0			
	Untreated	None	0			
	Wild Type	Focal lobular	1	1	0-1	
	Wild Type	None	0			
	Wild Type	None	0			
Cohort 2	Treated	None	0	0		0.2727
	Treated	None	0			
	Treated	None	0			
	Treated	None	0			
	Untreated	None	0	0		
	Untreated	None	0			
	Untreated	None	0			
	Untreated	None	0			
	Wild Type	Focal lobular and perivenular	2	1	0-2	
	Wild Type	None	0			
	Wild Type	None	0			
Cohort 3	Treated	None	0	1	0-3	0.3570
	Treated	Focal lobular and perivenular	2			
	Treated	Dense perivenular aggregate	3			
	Treated	None	0			
	Treated	Focal lobular	1			
	Untreated	None	0	0	0-1	
	Untreated	None	0			
	Untreated	None	0			
	Untreated	None	0			
	Untreated	Focal lobular	1			
	Wild Type	Focal lobular	1	1	0-1	
	Wild Type	Focal lobular	1			
	Wild Type	None	0			
Cohort 4	Treated	Focal lobular inflammation	1	1	0-2	0.2422
	Treated	Patchy lobular inflammation	1			
	Treated	No significant inflammation	0			
	Treated	Dense central inflammation and scattered lobular inflammation	1			
	Treated	No significant inflammation	0			
	Treated	Perivenular and scattered lobular inflammation	2			
	Treated	Possibly real central inflammation (PAS, looks like it is surrounding hepatocytes)	1			
	Treated	Scattered lobular inflammation	1			
	Untreated	No significant inflammation	0	2	0-3	
	Untreated	No significant inflammation	0			
	Untreated	Focal dense periportal aggregate (seen on PAS), and scattered lobular inflammation in minute foci	1			
	Untreated	Focal dense periportal aggregate and rare lobular inflammation	1			
	Untreated	Dense perivenular aggregate and rare lobular inflammation	3			
	Untreated	Very dense and large perivenular aggregate. Rare lobular inflammation	3			
	Untreated	Perivenular inflammation	2			
	Untreated	Multifocal perivenular inflammation	2			
	Untreated	Focal perivenular and focal lobular inflammation	2			
	Wild Type	Dense perivenular aggregate	3	2	1-3	
	Wild Type	Focal lobular inflammation	1			
	Wild Type	Focal notable dense perivenular aggregate and isolated focus of lobular inflammatory cells	3			
	Wild Type	Rare lobular inflammation	1			
	Wild Type	Rare lobular inflammation	1			

Table S5.

AAV gene therapy is not associated with inflammation in treated mice of all cohorts.

		Hematoxylin and Eosin		
		Nuclei Morphology	Hepatocyte Morphology	Steatosis
Cohort 4	Treated	Rare nuclear glycogen inclusions	Variably expanded by cytoplasmic glycogen, interspersed by hepatocytes WNL	None
	Treated	Rare nuclear glycogen inclusions, with one odd looking eosinophilic inclusion (H&E)	Variably expanded by cytoplasmic glycogen, interspersed by hepatocytes WNL	None
	Treated	Rare nuclear glycogen inclusions, with one odd looking eosinophilic inclusion (H&E)	Variably expanded by cytoplasmic glycogen, interspersed by hepatocytes WNL	None
	Treated	WNL	Variably expanded by cytoplasmic glycogen, interspersed by hepatocytes WNL	None
	Treated	Very rare glycogenated nuclei	Variably expanded by cytoplasmic glycogen, interspersed by hepatocytes WNL	None
	Treated	Rare nuclear glycogen inclusions	Variably expanded by cytoplasmic glycogen, interspersed by hepatocytes WNL	None
	Treated	Rare nuclear glycogen inclusions	Variably expanded by cytoplasmic glycogen, interspersed by hepatocytes WNL	None
	Treated	Rare nuclear glycogen inclusions	Variably expanded by cytoplasmic glycogen, interspersed by hepatocytes WNL	None
	Untreated	Rare nuclear glycogen inclusions	Variably expanded by cytoplasmic glycogen	None
	Untreated	Numerous glycogenated nuclei	Variably expanded by cytoplasmic glycogen	None
	Untreated	Numerous glycogenated nuclei	Variably expanded by cytoplasmic glycogen	None
	Untreated	Rare nuclear glycogen inclusions	Variably expanded by cytoplasmic glycogen	None
	Untreated	Rare nuclear glycogen inclusions	Variably expanded by cytoplasmic glycogen, fragile hepatocytes with poor morphology in liver tip	None
	Untreated	Rare nuclear glycogen inclusions	Variably expanded by cytoplasmic glycogen	None
	Untreated	Numerous glycogenated nuclei	Variably expanded by cytoplasmic glycogen	None
	Untreated	Rare nuclear glycogen inclusions	Variably expanded by cytoplasmic glycogen	None
	Untreated	Rare nuclear glycogen inclusions	Variably expanded by cytoplasmic glycogen	None
	Wild Type	WNL	WNL	None
	Wild Type	WNL	WNL	None
	Wild Type	WNL	WNL	None
	Wild Type	WNL	WNL	None
	Wild Type	WNL	WNL	None

Table S6.

AAV gene therapy restores hepatocyte architecture in treated mice.
